# Hydrogen peroxide, nitric oxide and UV RESISTANCE LOCUS8 interact to mediate UV-B-induced anthocyanin biosynthesis in radish sprouts

**DOI:** 10.1038/srep29164

**Published:** 2016-07-12

**Authors:** Qi Wu, Nana Su, Xiaoyan Zhang, Yuanyuan Liu, Jin Cui, Yongchao Liang

**Affiliations:** 1 College of Life Sciences, Nanjing Agricultural University, Nanjing 210095, Jiangsu, PR China; 2 Ministry of Education Key Laboratory of Environment Remediation and Ecological Health, College of Environmental & Resource Sciences, Zhejiang University, Hangzhou, 310058, China

## Abstract

The cross talk among hydrogen peroxide (H_2_O_2_), nitric oxide (NO) and UV RESISTANCE LOCUS8 (UVR8) in UV-B-induced anthocyanin accumulation in the hypocotyls of radish sprouts was investigated. The results showed that UV-B irradiation significantly increased the anthocyanin accumulation and the expression of *UVR8*, and a similar trend appeared in radish sprouts subjected to cadmium, chilling and salt stresses regardless of light source. However, these responses disappeared under dark exposure. These results suggest that abiotic stress-induced anthocyanin accumulation and *UVR8* expression were light-dependent. Moreover, abiotic stresses all enhanced the production of H_2_O_2_ and exogenous H_2_O_2_ addition significantly increased the anthocyanin concentration and *UVR8* transcription, while these increases were severely inhibited by addition of dimethylthiourea (DMTU, a chemical trap for H_2_O_2_). It seems to suggest that H_2_O_2_ played an important role in the anthocyanin biosynthesis. Furthermore, addition of 0.5 mM sodium nitroprusside (SNP, a NO-releasing compound) substantially induced the anthocyanin accumulation, and H_2_O_2_-induced anthocyanin accumulation and *UVR8* expression were significantly suppressed by co-treatment with 2-phenyl-4,4,5,5-tetramethylimidazoline-3-oxide-1-oxyl (PTIO, a NO scavenger), which was parallel with the expression of anthocyanin biosynthesis-related transcription factors and structural genes. All these results demonstrate that both H_2_O_2_ and NO are involved in UV-B-induced anthocyanin accumulation, and there is a crosstalk between them as well as a classical UVR8 pathway.

Anthocyanins are plant secondary metabolites synthesized through the flavonoid pathway, generating the characteristic reddish, bluish, and purple hues, which contribute to flower pigmentation, attraction of pollinators and seed dispersers^1^. In addition, they are also important as antioxidant molecules to protect plant cells against damage by reactive oxygen species (ROS)^2^, allelopathy, or UV irradiation^3^, and the production of anthocyanins in autumn leaves reduces the risk of photo-oxidative damage and delays leaf senescence^4^.On the other hand, possessing valuable nutritional antioxidant activities, anthocyanins are recognized as compounds with anti-inflammatory and anticancer effect, and potential health-benefits, such as prevention of cardiovascular diseases and obesity^5^,^6^.

The biosynthesis of anthocyanins is through phenylpropanoid pathway, which is one of the best studied examples of secondary metabolism in higher plants, and most genes responsible for anthocyanin biosynthesis have been cloned and analyzed^7^,^8^,^9^,^10^. The anthocyanins are synthesized from phenylalanine which is subsequently catalyzed by PAL, CHS, CHI, F3H, DFR, ANS, LDOX and UFGT^11^, and the accumulation of anthocyanins is induced by the expression of these genes which are regulated by a ternary transcriptional complex (MBW complex) containing an R2R3-MYB-type transcription factor, a bHLH transcription factor, and a WD40 repeat (WDR) protein^12^,^13^,^14^. The MBW complex is highly organized, and each subunit plays a specific function such as binding to DNA, activation of expression of a target gene, or stabilization of the transcription factor complex^15^. The physical interactions of WDR and bHLH proteins with more specific MYB proteins determine the regulation of anthocyanin accumulation^13^,^14^. The MYB superfamily consists of more than 100 members in higher plants^11^, among which both anthocyanin pigment 1 (PAP1) and anthocyanin pigment 2 (PAP2) are redundant R2R3-MYB transcription factors and positive regulate the expression of anthocyanin biosynthesis-related genes^16^.

The induction of anthocyanin accumulation in vegetative tissues is often considered to be a response of plants to biotic or abiotic stress conditions, such as nutrient (nitrogen and phosphorus) deficiency, wounding, pathogen infection, water stress, and ultraviolet (UV) light^17^,^18^. Among these various environmental stimuli, UV-B is reported to be a main factor in anthocyanin accumulation, which is concomitant with up-regulation of MYB and biosynthetic genes^19^,^20^,^21^,^22^,^23^. UV RESISTANCE LOCUS8 (UVR8) is a photoreceptor that specifically mediates photomorphogenic responses to UV-B in plants^24^. UVR8 is required for 1) a UV-B-stimulated compensatory increase in epidermal leaf cell size, 2) normal progression of endoreduplication in response to UV-B, 3) stomatal differentiation and functions in plant acclimation, and 4) survival under solar UV^25^,^26^. It is also found that the expression of *UVR8* is up-regulated in plants grown under salt or osmotic stress^27^. Furthermore, the ectopic expression of *UVR8* causes pleiotropic effects on plant growth, such as reduced plant organ size and root growth, and increased accumulation of flavonoids^28^.

UVR8 is a homodimer in its ground state, and UV-B exposure results in its instantaneous monomerization (the active form) followed by interaction with CONSTITUTIVELY PHOTOMORPHOGENIC 1 (COP1), a major factor in UV-B signaling^29^,^30^,^31^. Recently, some studies^32^,^33^,^34^ found that constitutively active UVR8 variants elevated levels of anthocyanins but UVR8 deficient mutant had lower levels of anthocyanins, suggesting that UVR8 may be involved in regulation of the biosynthesis of anthocyanins. However, details in the primary mechanisms of the involvement of UVR8 in anthocyanin biosynthesis are still scant.

UV-B irradiation may induce the production of ROS^35^, among which H_2_O_2_ is a major species generated in plants^36^. Because H_2_O_2_ is relatively stable and diffusible through membrane, it is generally thought to serve as a signal molecule under various forms of abiotic stress^37^. It has been reported that H_2_O_2_ could regulate the biosynthesis of anthocyanins^38^,^39^, and mediate the regulatory effect of UVR8 in stomatal closure^40^. In addition, nitric oxide (NO) is regarded as a downstream gaseous molecule of H_2_O_2_ to mediate UV-B-induced stomatal closure^40^,^41^. Based on all these reports, we hypothesize that NO, H_2_O_2_ and UVR8 may interact to regulate UV-B-induced anthocyanin biosynthesis.

In the present study, cherry radish sprouts were chosen as the materials, which contain substantial amounts of antioxidants, vitamin C and health-promoting compounds such as glucosinolates and phenolic compounds^42^,^43^,^44^. Consumption of radish sprouts has been confirmed to reduce the risk of cancer and oxidative stress both *in vivo* and *in vitro*^45^,^46^,^47^. Furthermore, with red hypocotyls resulting from anthocyanin accumulation, cherry radish sprouts could provide visual evidence for the biosynthesis of anthocyanins, and consequently, the high concentration of anthocyanin accumulated in cherry radish sprouts could underpin their high antioxidant activity. Using radish sprouts, in the present paper we confirmed the involvement of the UVR8 pathway in UV-B-induced anthocynin accumulation. Moreover, our results demonstrated that H_2_O_2_-dependent production of NO interacted with UVR8 to regulate the biosynthesis of anthocyanins.

## Results

### UVR8 was involved in the biosynthesis of anthocyanins

**Figure 1:**
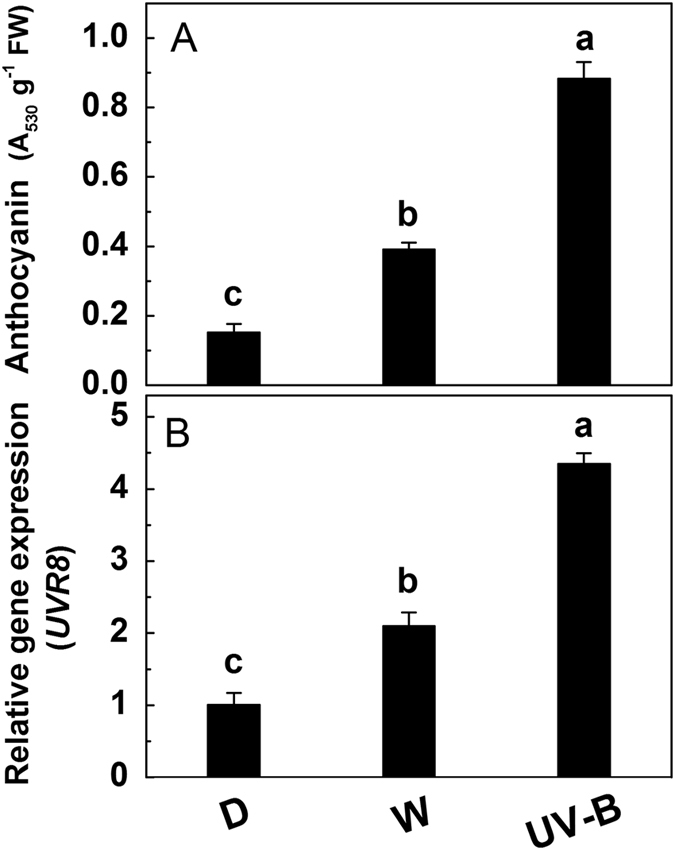
The concentration of anthocyanins (**A**) and the expression of *UVR8* (**B**) in the hypocotyls of radish sprouts under dark (D), white light (W) and UV-B. After 36 h dark incubation, the radish sprouts were then exposed to dark, white light or UV-B for another 36 h when the samples were collected and detected. The data are means ± SD of three independent experiments. Significance between experimental values was assessed by Duncan’s test (*P* < 0.05).

To determine the involvement of UVR8 in the biosynthesis of anthocyanins, the concentration of anthocyanin and the transcript level of *UVR8* in the hypocotyls of radish sprouts grown under dark, white light and UV-B were analyzed. As expected, compared with dark environment, white light significantly increased the concentration of anthocyanins, while the highest anthocyanin concentration (2 times greater than that under white light) was observed after 24 h UV-B irradiation (Fig. 1A). Similarly, the expression of *UVR8* was significantly induced by UV-B, which was 2-fold that under white light (Fig. 1B).

**Figure 2:**
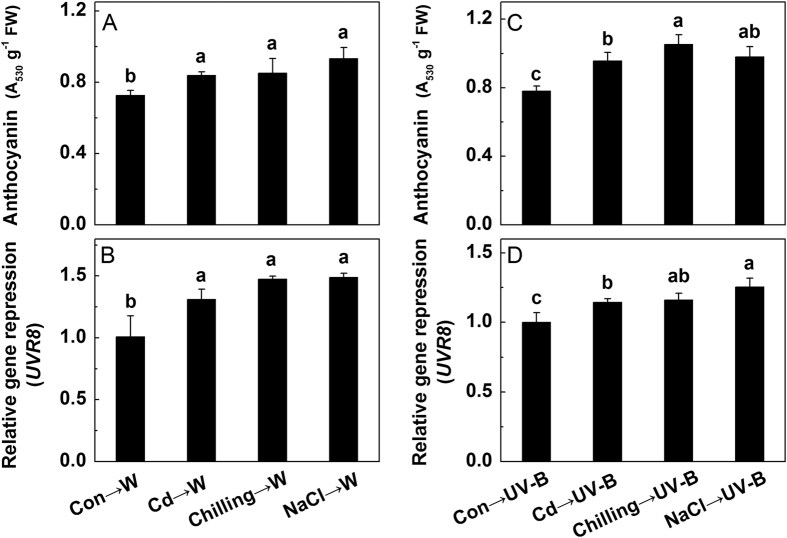
The concentration of anthocyanins (**A,C**) and the expression of *UVR8* (**B,D**) in the hypocotyls of radish sprouts under different treatments. After 24 h dark culture, sprouts were subjected to cadmium (Cd), chilling, NaCl or nothing (Con) for 12 h still under dark, then these sprouts were transferred to white light or UV-B for 24 h. Values are the means ± standard error of three independent experiments with at least three replicates for each. Measurements in the same column followed by different letters are significantly different at *P* < 0.05 level by Duncan’s test.

Previous studies show that UVR8 is involved in salt or osmotic stress conditions^17^. To investigate whether there is a relationship between UVR8 and abiotic stresses in the biosynthesis of anthocyanins, we compared the anthocyanin accumulation and *UVR8* expression under control condition and abiotic stress. The results showed that regardless of white light or UV-B irradiation, cadmium, chilling and NaCl treatments all induced significant increases in anthocyanin accumulation and the transcript abundance of *UVR8*, compared with those under control (Fig. 2).

**Figure 3:**
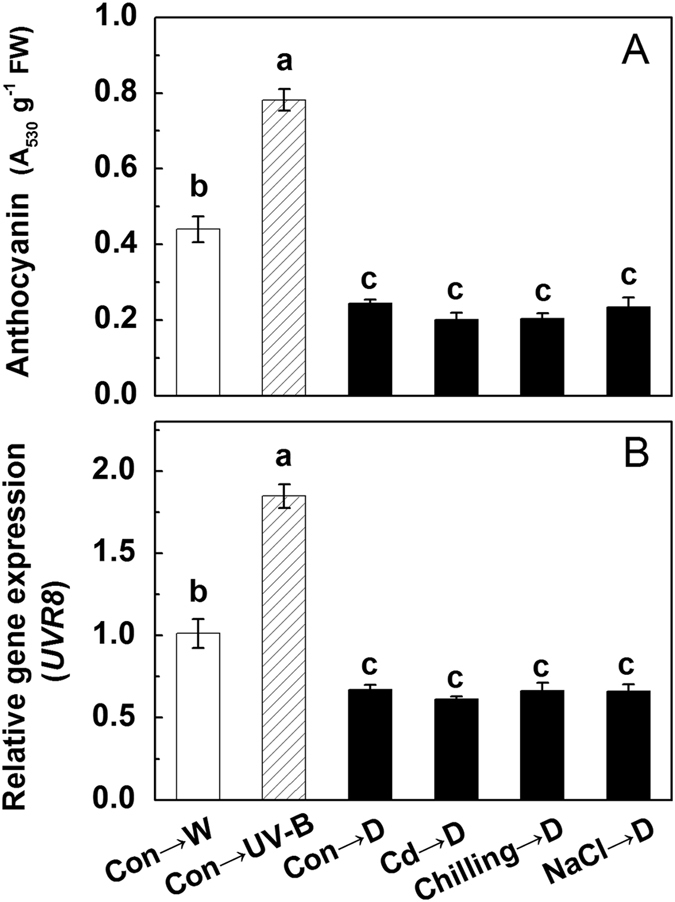
The concentration of anthocyanins (**A**) and the expression of *UVR8* (**B**) in the hypocotyls of radish sprouts under different treatments. After 24 h dark culture, sprouts were subjected to nothing (Con), cadmium (Cd), chilling or NaCl for 12 h still under dark, then these sprouts were transferred to white light, UV-B or still dark for 24 h. The data represent means ± SD of three independent experiments. Significance between experimental values was assessed by Duncan’s test (*P* < 0.05).

However, it remains unknown whether abiotic stress-induced anthocyanin accumulation and *UVR8* expression are light-dependent. To test this, we repeated the above experiment under a dark environment. In contrast to exposure to light, abiotic stresses under dark had no effects on the anthocyanin accumulation and the expression of *UVR8* (Fig. 3), suggesting that light was a necessary factor in stress-induced anthocyanin accumulation and *UVR8* expression.

### H_2_O_2_ was involved in UV-B-induced biosynthesis of anthocyanins

**Figure 4:**
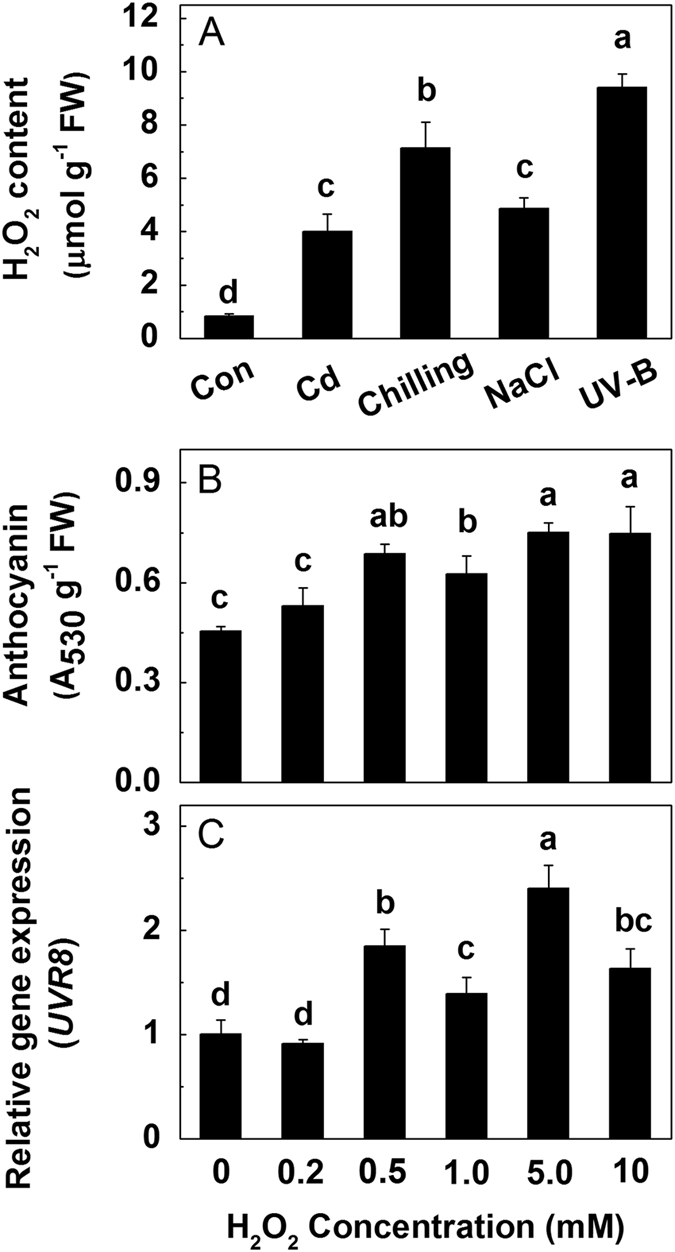
The H_2_O_2_ concentration in the hypocotyls of radish sprouts subjected to Cd, chilling, NaCl or UV-B (**A**). After 24 h dark incubation, the radish sprouts were then subjected to different abiotic stresses for 12 h, and then they were exposed to white light for another 24 h. The anthocyanin accumulation (**B**) and the expression of *UVR8* (**C**) in the hypocotyls of radish sprouts treated with increasing exogenous H_2_O_2_. After 24 h dark incubation, the radish sprouts were then subjected to H_2_O_2_ with different concentrations for 12 h, and then they were exposed to white light for another 24 h. The data represent means ± SD of three independent experiments. Significance between experimental values was assessed by Duncan’s test (*P* < 0.05).

To identify the role of H_2_O_2_ under different stresses and the relationship between H_2_O_2_ and the anthocyanin accumulation, the H_2_O_2_ concentrations under stresses and the effects of H_2_O_2_ on anthocyanin accumulation and *UVR8* expression were tested. Similarly, cadmium, chilling and salt treatments as well as UV-B irradiation all significantly increased the H_2_O_2_ concentration, with the maximum observed under UV-B, which is about 20 times as great as that of control (Fig. 4A). The accumulation of anthocyanins was positively correlated with the H_2_O_2_ concentration, though no significant difference was noted between control and the treatment with 0.2 mM H_2_O_2_ (Fig. 4B). The expression levels of *UVR8* were also up-regulated by H_2_O_2_ ranging from 0.5 to 10 mM, while no positive correlation between them was observed (Fig. 4C). Therefore, 0.5 mM H_2_O_2_ was selected as the appropriate treatment concentration in the following experiments.

**Figure 5:**
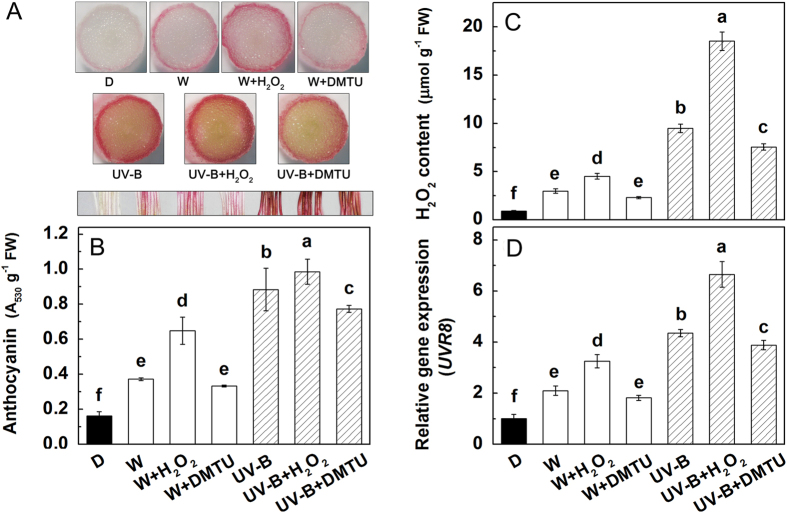
Effects of exogenous addition of H_2_O_2_ or DMTU on the pigmentation of hypocotyls (**A**), anthocyanin accumulation (**B**), H_2_O_2_ concentration (**C**) and *UVR8* expression (**D**). After 36 h dark incubation, the radish sprouts were then subjected to H_2_O_2_ or DMTU under white light or UV-B for 24 h. The data represent means ± SD of three independent experiments. Significance between experimental values was assessed by Duncan’s test (*P* < 0.05).

Addition of 0.5 mM H_2_O_2_ significantly enhanced the pigmentation in the hypocotyls of radish sprouts, regardless of exposure to white light or UV-B, while addition of DMTU (dimethylthiourea, a chemical trap for H_2_O_2_) effectively inhibited UV-B-induced anthocyanin accumulation (Fig. 5A,B). The concentration of H_2_O_2_ and the level of *UVR8* both showed similar trends with the accumulation of anthocyanins (Fig. 5C,D).

### NO was involved in UV-B-induced biosynthesis of anthocyanins

To verify the involvement of NO in activating anthocyanin biosynthesis, the endogenous NO levels and anthocyanin concentrations in response to various concentrations of exogenous SNP (sodium nitroprusside, a NO-releasing compound) were determined. As shown in Fig. S1, the endogenous levels of NO almost increased linearly with increasing SNP concentrations (Fig. S1A). The anthocyanin accumulation was significantly increased by addition of 0.5 mM SNP, and decreased afterward with further increasing of SNP (Fig. S1B).

### The relationship underlying NO, H_2_O_2_ and UVR8 in anthocyanin accumulation

**Figure 6:**
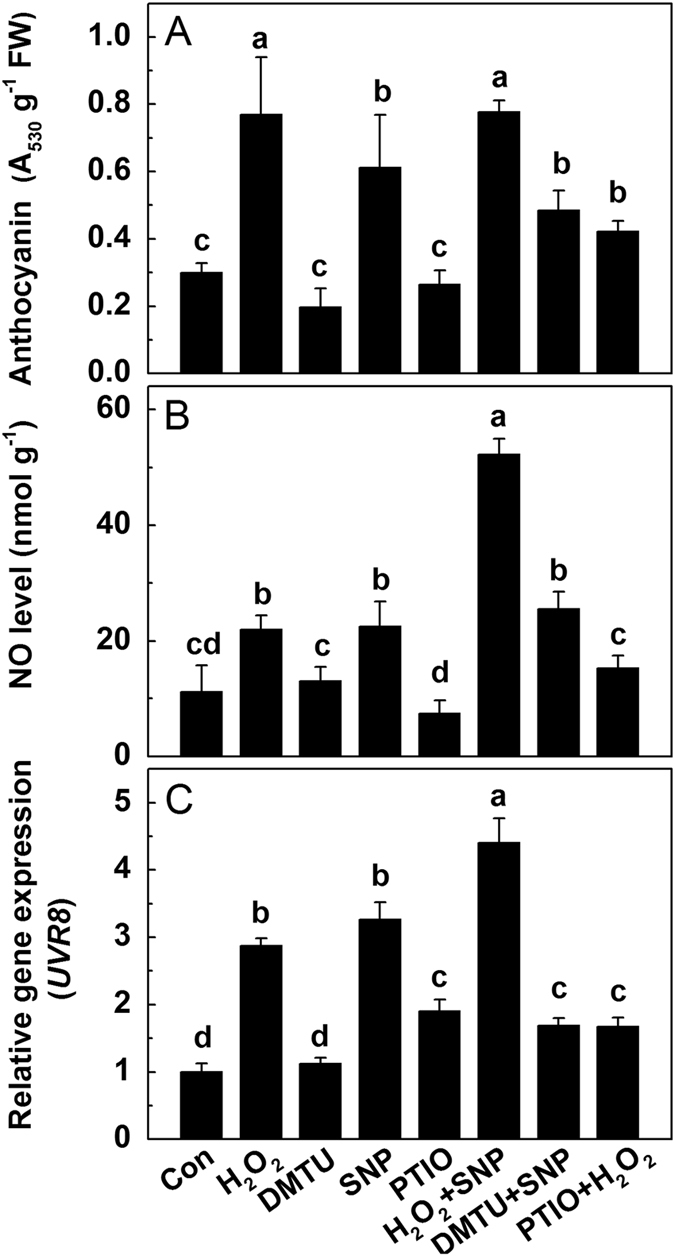
The anthocyanin accumulation (**A**), endogenous NO level (**B)** and *UVR8* expression (**C**) under different treatments. After 36 h dark incubation, the radish sprouts were then subjected to H_2_O_2_, DMTU, SNP, PTIO or their combination under white light for 24 h. The data represent means ± SD of three independent experiments. Significance between experimental values was assessed by Duncan’s test (*P* < 0.05).

Similar to the results above, addition of H_2_O_2_, SNP or their combination all considerably enhanced the anthocyanin biosynthesis, while in comparison with SNP, co-treatment with DMTU slightly offset this increase, though it was not statistically significant. Moreover, H_2_O_2_-induced increase of anthocyanins was dramatically suppressed by co-treatment with PTIO (Fig. 6A). The NO level and *UVR8* expression were both consistent with this trend, except that the combination of DMTU and SNP significantly inhibited SNP-induced increase of *UVR8* expression (Fig. 6B,C).

**Figure 7:**
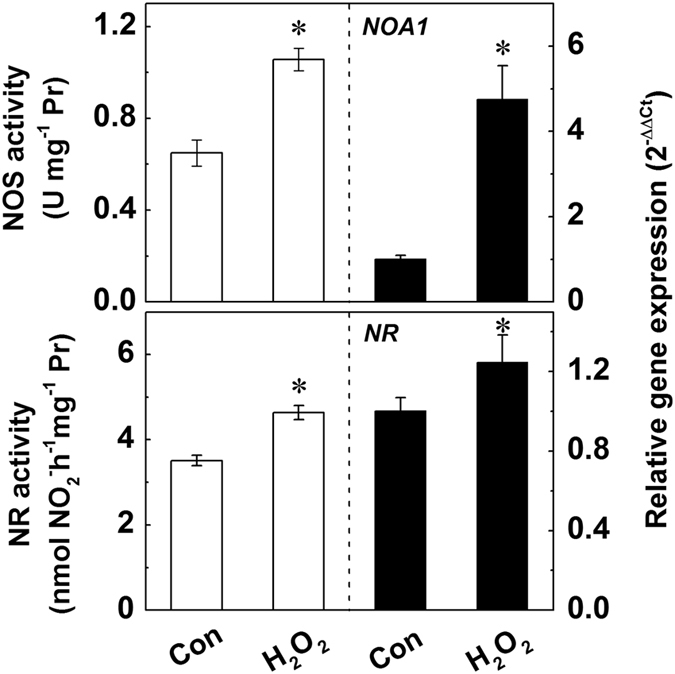
Effects of H_2_O_2_ on the activities of NOS and NR and the expression levels of *NOA1* and *NR*. After 24 h dark incubation, the radish sprouts were then subjected to H_2_O_2_ for 12 h, and then they were exposed to white light for another 24 h. The data represent means ± SD of three independent experiments. Significance between experimental values was assessed by Duncan’s test (*P* < 0.05).

Considering that H_2_O_2_ induces the generation of NO which is involved in many plant physiologic responses, we examined the effect of H_2_O_2_ on the enzymes and genes responsible for NO generation. As reported previously^48^,^49^, H_2_O_2_ significantly enhanced the activities of NOS and NR, and up-regulated the expression of *NOA1* and *NR* by 5 and 1.2-fold, respectively (Fig. 7).

**Figure 8:**
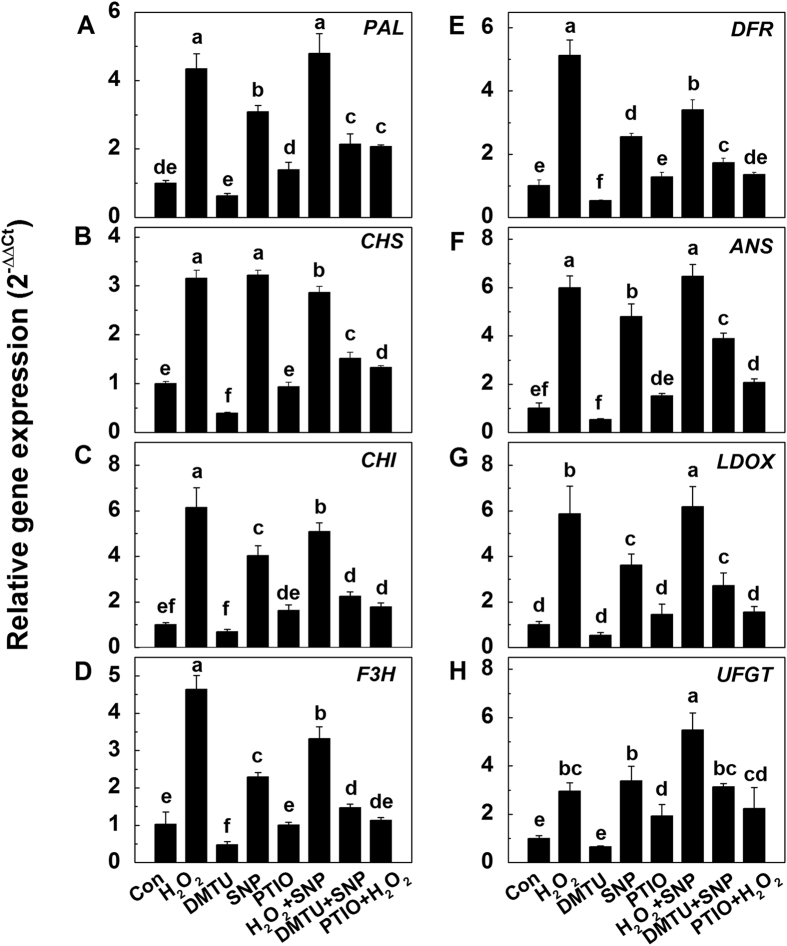
The transcript levels of anthocyanin biosynthesis-related genes *PAL* (**A**), *CHS* (**B**), *CHI* (**C**), *F3H* (**D**), *DFR* (**E**), *ANS* (**F**), *LDOX* (**G**) and *UFGT* (**H**) under different treatments. After 36 h dark incubation, the radish sprouts were then subjected to H_2_O_2_, DMTU, SNP, PTIO or their combination under white light for 24 h. The data represent means ± SD of three independent experiments. Significance between experimental values was assessed by Duncan’s test (*P* < 0.05).

Finally, the expression levels of transcription factors and structural genes responsible for anthocyanin biosynthesis were further determined by qRT-PCR. The relative expressions of *COP1*, *PAP1* and *PAP2* displayed similar trends. Compared with the control, H_2_O_2_, SNP and their combination all significantly up-regulated the transcript levels, while DMTU and PTIO had no effects on the transcription of *COP1*, *PAP1* and *PAP2*, with the exception of the considerable decrease of *COP1* under DMTU treatment. Compared with H_2_O_2_, co-treatment with PTIO significantly down-regulated the transcripts of *COP1*, *PAP1* and *PAP2*. Compared with SNP, combined treatment with DMTU and SNP decreased the transcription of *COP1* and *PAP1* (Fig. S3). The transcript levels of anthocyanin-biosynthesis-related structural genes (*PAL*, *CHS*, *CHI*, *F3H*, *DFR*, *ANS*, *LDOX* and *UFGT*) displayed the similar trends to the transcription factors (Fig. 8). In detail, H_2_O_2_, SNP and their combination significantly up-regulated the transcript levels of these genes, while H_2_O_2_ + PTIO and SNP + DMTU substantially suppressed their expressions.

## Discussion

Since its initial discovery in 2002^37^, UVR8 has been the focus of recent attention due to numerous reports of its regulatory effects on UV-B signaling pathway and stress responses in plants^50^,^51^. However, the exploration of UVR8 function *in vivo* is still at an early stage and information about UVR8 is virtually derived from studies with Arabidopsis. Therefore its physiological roles in other diverse species remain poorly understood^52^. In this study, we explored the cross talk among H_2_O_2_, NO and UVR8 in regulating anthocyanin accumulation in the hypocotyls of radish sprouts.

Similar to previous reports in peach, strawberry and lettuce^53^,^54^,^55^, compared with white light and dark, UV-B considerably increased the anthocyanin accumulation in the hypocotyls of radish sprouts (Fig. 1A). Though it has been found that UV-B exposure induces the conversion of UVR8 from a homodimer to a monomeric which will interact with transcription factor COP1^40^,^52^, the *UVR8* expression showed the consistent trend with the anthocyanin accumulation (Fig. 1B), suggesting that as a UV-B photoreceptor, UVR8 is likely to be involved in mediating anthocyanin biosynthesis. This hypothesis is supported by a recent study by Mao *et al.*^56^ who found that PeUVR8 in *Populus euphratica* could increase the anthocyanin accumulation in the Arabidopsis uvr8 mutant.

Apart from UV-B, other abiotic stresses could also induce the biosynthesis of anthocyanins, such as high light intensity^57^, low temperature^58^, and nutrient deficiency^59^. In this study, we also observed that the anthocyanin accumulation was significantly increased by three typical abiotic stresses, cadmium, chilling and salt, regardless of supply with white light or UV-B (Fig. 2A,C). Meanwhile, the expression of *UVR8* showed a similar trend (Fig. 2B,D), which was in line with the study of Fasano *et al.*^27^, suggesting that UVR8 was involved in abiotic stresses, and there was a potential relationship between anthocyanins and UVR8. By contrast, these increases were not observed under dark (Fig. 3), illustrating that abiotic stress-induced anthocyanin accumulation and increased expression of *UVR8* were light-dependent.

Taking into consideration the importance of H_2_O_2_ in abiotic stresses and anthocyanin accumulation, we speculated that H_2_O_2_ may be a conjunctive point between anthocyanins and UVR8. As expected, compared with control, the concentration of H_2_O_2_ under various abiotic stresses (Cd, chilling, NaCl and UV-B) was increased significantly (Fig. 4A), and exogenous addition of H_2_O_2_ (0.5–10 mM) enhanced both the anthocyanin accumulation and the expression of *UVR8* (Fig. 4B,C). Furthermore, UV-B-induced anthocyanin accumulation, H_2_O_2_ concentration and *UVR8* expression were all suppressed by addition of DMTU (an inhibitor of H_2_O_2_), although DMTU did not affect these factors under white light (Fig. 5), which might be due to the low H_2_O_2_ accumulation under normal growth condition. These results imply that UV-B irradiation induces more production of H_2_O_2_, which further up-regulates UV-B-induced *UVR8* expression to modulate the biosynthesis of anthocyanins.

Several previous studies showed that NO acts as downstream gaseous signal molecule of H_2_O_2_ to regulate the stomatal closure^40^,^60^, which prompted us to further investigate the role of NO in anthocyanin accumulation and the relationship among NO, H_2_O_2_ and UVR8. The endogenous NO concentration in radish sprouts showed an almost linear increase with the concentration of SNP (the donor of NO) (Fig. S1A), with 0.5 mM SNP significantly increased the anthocyanin accumulation (Fig. S1B). Furthermore, we observed that H_2_O_2_-induced increases of anthocyanin accumulation, NO concentration and *UVR8* expression were all considerably restrained by co-treatment with PTIO (a scavenger of NO) (Fig. 6).

In animals, NO is synthesized through NO synthase (NOS)^61^ which has also been detected widely in plants, and inhibitors of mammalian NOS also have the ability to inhibit the generation of NO in plants, though no archetypal NOS-encoding gene(s) have been isolated in higher plants so far^62^,^63^. The nitric oxide associated1 (NOA1), reported to encode a protein with NOS activity, has been shown to be a GTPase playing a role in binding RNA/ribosomes^64^. In addition, it has been reported that NOA1-mediated NO production was involved in plant responses against salinity stress^65^ and pathogens^66^. In plants the NO production can also be generated via nitrate reductase (NR), a critical enzyme responsible for nitrate assimilation^67^,^68^. In the present study, the enzyme activity and gene expression related to NO biosynthesis were all significantly enhanced by addition of H_2_O_2_ (Fig. 7). All these results suggest that irradiation with UV-B enhanced the production of H_2_O_2_, which increased the level of NO to further magnify UV-B-induced expression of *UVR8* to regulate the anthocyanin biosynthesis. This hypothesis was further verified by the expressions of transcription factors and structural genes responsible for anthocyanin biosynthesis under various treatments (Fig. S3 and 8).

**Figure 9:**
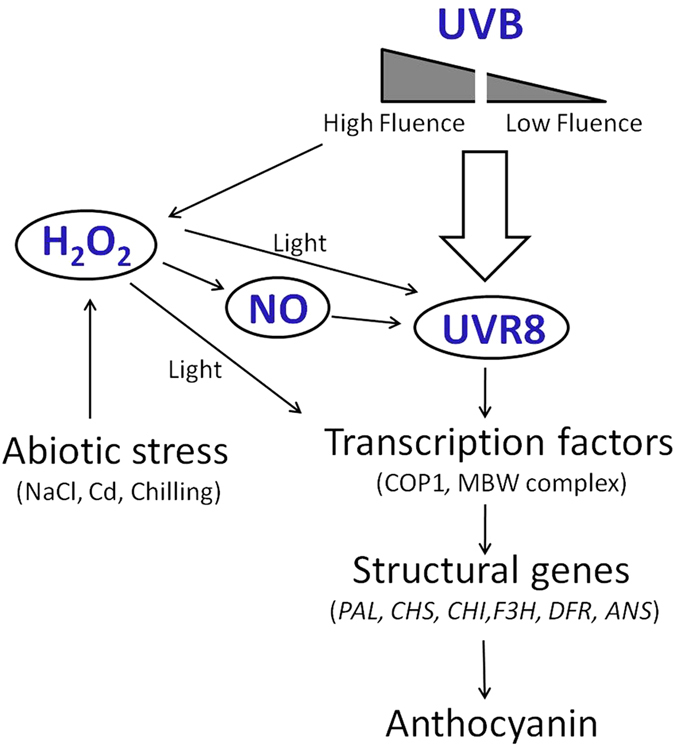
Model of H_2_O_2_ and NO participating in UV-B-induced anthocyanin biosynthesis. In a identified path, UVR8, the photoreceptor of UV-B, firstly perceives the signal from low-intensity-UV-B irradiation, and then initiates the transcription factors (COP1, MBW complex) which further up-regulates the expression of anthocyanin biosynthesis-related structural genes (*PAL*, *CHS*, *CHI*, *F3H*, *DFR*, *ANS*), finally to enhance the accumulation of anthocyanins. Besides the signal, high-intensity-UVB could also act as an environmental stress which, to some extent, induces the production of H_2_O_2_ which could also be induced by abiotic stress. H_2_O_2_ may promote the biosynthesis of anthocyanins through three pathways: 1) H_2_O_2_ interacts with UVR8, which is light-dependent; 2) H_2_O_2_ firstly stimulates the production of NO that interacts with the UVR8; 3) H_2_O_2_ up-regulates the expression of transcription factors directly, which is also light-dependent.

The UVR8-dependent UV-B signaling pathway operates at low fluence rates to initiate classical UV-B photomorphogenic responses, such as the induction of flavonoid biosynthesis^69^. Therefore, the common pathway of low-intensity-UV-B-induced anthocyanin accumulation may be that UVR8 perceives the UV-B signal, and then transforms it into monomers^70^,^71^, which interact with the E3 ubiquitin ligase CONSTITUTIVELY PHOTOMORPHOGENIC1 (COP1)^72^ to activate gene expression involved in anthocyanin biosynthesis in plants. However, it was reported that both low and high fluence of UV-B could activate UVR8^73^. In this study, we reported that both H_2_O_2_ and NO were involved in high-intensity-UV-B-induced anthocyanin accumulation. In detail, high fluence of UV-B or abiotic stress-induced production of H_2_O_2_ could regulate the biosynthesis of anthocyanins through the following three pathways: 1) H_2_O_2_ directly up-regulates the expression of *UVR8*, which is light-dependent; 2) H_2_O_2_ increases the concentration of NO which magnifies the effects of UV-B on *UVR8* transcription; and 3) H_2_O_2_ influences the redox which changes the expressions of anthocyanin biosynthesis-related transcription factors, and this process is also light-dependent (Fig. 9). In this study, the UV-B lamps were used as the light source and the spectrum ranges from 280 nm to 360 nm with its peak of 306 nm (Fig. S4), which is a related broad spectrum of light including UV-A. Therefore, the UV-B induced anthocyanin biosynthesis might be partly due to the broad spectrum of light. As the spectrum peak of UV-B lamps is 306 nm, we estimated that in this study, the enhancement of anthocyanin was mainly induced by UV-B, which should be further investigated under a narrow band of UV-B. In addition, the emergence of LED UV-B would facilitate the related researches.

## Materials and Methods

### Plant materials and treatments

Seeds of radish (*Raphanus sativus* L cv. Yanghua) were soaked in deionized water for about 12 h, and then put on moist gauze to germinate for 24 h. Uniform-sized germinated seeds were selected and sown into cases containing1/4 strength Hoagland’s solution and covered with gauze. These cases were covered with gauze and were maintained in an incubator in dark at 25 °C for 24 or 36 h. Then the sprouts were treated grown in growth chambers equipped with LED white light (Safe Instrument Experimental Factory, Zhejiang, China) or UV-B lamps (Sankyo Denki, Shinagawa, Tokyo, Japan) which emit ultraviolet rays between 280 nm and 360 nm with its peak of 306 nm (Fig. S4). The light intensity of white light was set at 50 ± 5 μmol·m^−2^·s^−1^, while the UV-B dose was set at 10 W·m^−2^ measured with a pocket UV light meter (UV-340 A, Lutron, Taiwan). The temperature was 25 °C and the relative humidity (RH) was 65–75%.

### Anthocyanin analysis

The total anthocyanin concentration in the radish hypocotyls was determined according to the method developed by Zhou *et al.*^74^, which involves measuring the absorbance (530) of extracts. Briefly, samples (0.5 g) were incubated in 5 mL of 1% HCl in methanol at room temperature in the dark for 24 h. Tubes were shaken with a vortex mixer every 6 hours. The anthocyanins in the aqueous phase were then quantified spectrophotometrically (A_530_ − 0.25 × A_657_) (UV-5200 spectrophotometer, Shanghai Metash Instruments Co., Ltd, Shanghai, China).

### Observation of the hypocotyls cross section

The hypocotyls of radish sprouts were transected with a blade and then observed under a light microscope (Model Stemi 2000-C; Carl Zeiss, Germany) and photographed on a color film (Powershot A620, Canon Photo Film, Japan).

### Quantitative and Real-time RT-PCR analysis

Total RNA was isolated from root tissues using Trizol extraction reagent (Invitrogen, Gaithersburg, MD, USA) and the RNA purity was verified by the ratio (>1.9) of 260/280 nm absorbance. DNA-free total RNA (8 μl) from different treatments was used for first-strand cDNA synthesis in a 20 μl reaction volume (Thermo Scientific, MD, Lithuania) according to the manufacturer’s instructions. Real-time quantitative PCR reactions were performed using a Mastercycler^®^ eprealplex real-time PCR system (ABI 7500, MD, USA) with Bestar^®^ SybrGreen qPCR mastermix (DBI, Bioscience Inc., Germany) in a 20 μl reaction volume according to the user manual.

PCR primers targeting *actin*, *UVR8, COP1, NOA1, NR, PAL, CHS, CHI, F3H, DFR, LDOX, ANS, UFGT, PAP1* and *PAP2* were designed using Primer Express^®^ version 3.0 (Applied Biosystems). In order to design the UVR8 primer, the UVR8 mRNA of Arabidopsis was blasted against the radish data base (RadishBase), and a similar mRNA (Unigene ID: UN17090, Length: 1690) was obtained. The similarity is 90%. As UVR8 proteins occur widely among plant species and are well conserved^72^, the products from this prime were thought to be UVR8. All primers (Table S1) were synthesized by Genewiz Bio-engineering Ltd. Company (Suzhou, China). The relative expression levels were presented as values relative to that of corresponding control sample at the indicated time, after normalization to *actin* transcript levels.

### Determination the activities of NR and NOS

The activity of NR was determined as described by Sun *et al.*^75^. The hypocotyls (0.5 g) were homogenized with a mortar and pestle on ice with 5 ml of extract buffer containing 50 mM HEPES-KOH (pH 7.5), 5% glycerol (v/v), 10 mM MgCl_2_, 1 mM dithiothreitol (DTT), 1 mM phenylmethylsulfonyl fluoride (PMSF), and 10 μM flavin adenine dinucleotide (FAD). The extract was centrifuged at 13 000 g for 20 min at 4 °C.

The activity of NR was measured immediately by mixing 250 μl of supernatant with 250 μl prewarmed (25 °C) assay buffer containing 50 mM HEPES-KOH (pH 7.5), 10 mM MgCl_2_, 1 mM DTT, 2 mM KNO_3_ and 200 μM NADH. The reaction was started by adding assay buffer, incubated at 30 °C for 30 min and then stopped by adding 50 μl 0.5 M Zn-acetate. The nitrite produced was measured colorimetrically at 540 nm after adding 1 ml of 1% sulfanilamide in 3 M HCl plus 1 ml of 0.02% N-(1-naphthyl) ethylenediamine in 0.2 M HCl.

To determine NOS activity, the total protein was extracted using the buffer containing 100 mM HEPES-KOH (pH 7.5), 1 mM EDTA, 10% glycerol (v/v), 5 mM DTT, 0.5 mM PMSF, 0.1% Triton X-100 (v/v), 1% polyvinylpyrrolidone (PVP) and 20 μM FAD. NOS activity was then measured after centrifugation at 13 000 g for 20 min at 4 °C according to Gonzalez *et al.*^76^. Briefly, NOS activity was detected in1 ml of reaction mixture containing 100 mM phosphate buffer(pH 7.0), 1 mM L-Arg, 2 mM MgCl_2_, 0.3 mM CaCl_2_, 4 μM BH_4_, 1 μM FAD, 1 μM flavin mononucleotide (FMN), 0.2 mM DTT, 0.2 mM NADPH, and 200 μl of protein extract. The decrease in absorbance as a result of NADPH consumption was determined at 340 nm for 5 min. NOS activity was calculated using the extinction coefficient of NADPH (e = 6.22 mM^−1^ cm^−1^).

### Detection of H_2_O_2_ concentration

The H_2_O_2_ concentration was measured colorimetrically as described by Hung^77^. H_2_O_2_ was extracted by homogenizing hypocotyl tissue with phosphate buffer (50 mM, pH 6.5) containing 1 mM hydroxylamine. The homogenate was centrifuged at 6,000 g for 25 min. To determine H_2_O_2_ concentration, the extracted solution was mixed with 0.1% titanium chloride in 20% (v/v) H_2_SO_4_. The mixture was then centrifuged at 6,000 g for 25 min. The absorbance was measured at 410 nm. The NO concentration was calculated by comparing to against a standard curve of H_2_O_2_.

### Quantification of NO concentration

Nitric oxide concentration was determined using the method described by Hu *et al.*^78^ and Zhou *et al.*^74^ with slight modifications. Samples (1.0 g) were ground in a mortar and pestle in 8 ml of 50 mM cool acetic acid buffer (pH 3.6, containing 4% zinc diacetate). The homogenates were centrifuged at 10,000 g for 15 min at 4 °C. The supernatant was collected. The pellet was washed by 1 ml of extraction buffer and centrifuged as before. The two supernatants were combined and 0.1 g of charcoal was added. After vortex and filtration, the filtrate was leached and collected. The mixture of 1.5 ml of filtrate with1.5 ml of the Greiss reagent was incubated at room temperature for 30 min. Absorbance was determined at 540 nm. The NO concentration was calculated against a standard curve of NaNO_2_.

### Statistical analyses

Values presented are means ± standard deviation (SD) of three replicates. Data was subjected to analysis of variance (ANOVA), and mean values were compared by Duncan’s multiple range test (p < 0.05). All the statistical analyses were performed using SPSS 19.0 for Windows.

## Additional Information

**How to cite this article**: Wu, Q. *et al.* Hydrogen peroxide, nitric oxide and UV RESISTANCE LOCUS8 interact to mediate UV-B-induced anthocyanin biosynthesis in radish sprouts. *Sci. Rep.* **6**, 29164; doi: 10.1038/srep29164 (2016).

## Supplementary Material

Supplementary Information

## References

[b1] Yoshida K., Ma D. W. & Constabel C. P. The MYB182 protein down-regulates proanthocyanidin and anthocyanin biosynthesis in poplar by repressing both structural and regulatory flavonoid genes. Plant Physiol. 167, 693–710 (2015). 25624398 10.1104/pp.114.253674 PMC4348771

[b2] Gould K. S., Mckelvie J. & Markham K. R. Do anthocyanins function as antioxidants in leaves? Imaging of H_2_O_2_ in red and green leaves after mechanical injury. Plant Cell. Environ. 25, 1261–1269 (2002).

[b3] Treutter D. Significance of flavonoids in plant resistance and enhancement of their biosynthesis. Plant Biol. 7, 581–591 (2005). 16388461 10.1055/s-2005-873009

[b4] Schaberg P., Murakami P., Turner M., Heitz H. & Hawley G. Association of red coloration with senescence of sugar maple leaves in autumn. Trees. 22, 573–578 (2008).

[b5] He J. & Giusti M. M. Anthocyanins: natural colorants with health-promoting properties. Annu. Rev. Food. Sci. T. 1, 163–187 (2010). 10.1146/annurev.food.080708.100754 22129334

[b6] Tsuda T. Regulation of adipocyte function by anthocyanins; possibility of preventing the metabolic syndrome. J. Agric. Food. Chem. 56, 642–646 (2008). 18211021 10.1021/jf073113b

[b7] Holton T. A. & Cornish E. C. Genetics and biochemistry of anthocyanin biosynthesis. Plant Cell. 7, 1071–1083 (1995). 12242398 10.1105/tpc.7.7.1071 PMC160913

[b8] Forkmann G. & Martens S. Metabolic engineering and applications of flavonoids. Curr Opin Biotechnol. 12, 155–160 (2001). 11287230 10.1016/s0958-1669(00)00192-0

[b9] Shirley B. W. Flavonoid biosynthesis. A colorful model for genetics, biochemistry, cell biology, and biotechnology. Plant Physiol. 126, 485–493 (2001). 11402179 10.1104/pp.126.2.485 PMC1540115

[b10] Koes R., Verweij W. & Quattrocchio F. Flavonoids: a colorful model for the regulation and evolution of biochemical pathways. Trends Plant Sci. 10, 236–242 (2005). 15882656 10.1016/j.tplants.2005.03.002

[b11] Sakuta M. Diversity in plant red pigments: anthocyanins and betacyanins. Plant Biotechnol.Rep. 8, 37–48 (2014).

[b12] Baudry A. *et al.* TT2 TT8, and TTG1 synergistically specify the expression of BANYULS and proanthocyanidin biosynthesis in Arabidopsis thaliana. Plant J. 39, 366–380 (2004). 15255866 10.1111/j.1365-313X.2004.02138.x

[b13] Broun P. Transcriptional control of flavonoid biosynthesis: a complex network of conserved regulators involved in multiple aspects of differentiation in Arabidopsis. Curr Opin Plant Biol. 8, 272–279 (2005). 15860424 10.1016/j.pbi.2005.03.006

[b14] Ramsay N. A. & Glover B. J. MYB-bHLH-WD40 protein complex and the evolution of cellular diversity. Trends Plant Sci. 10, 63–70 (2005). 15708343 10.1016/j.tplants.2004.12.011

[b15] Hichri I. *et al.* Recent advances in the transcriptional regulation of the flavonoid biosynthetic pathway. J Exp Bot. 62, 2465–2483 (2011). 21278228 10.1093/jxb/erq442

[b16] Gonzalez A., Zhao M., Leavitt J. M. & Lloyd A. M. Regulation of the anthocyanin biosynthetic pathway by the TTG1/bHLH/Myb transcriptional complex in Arabidopsis seedlings. Plant J. 53, 814–827 (2008). 18036197 10.1111/j.1365-313X.2007.03373.x

[b17] Dixon R. & Paiva N. Stress-induced phenylpropanoid metabolism. Plant Cell. 7, 1085–1097 (1995). 12242399 10.1105/tpc.7.7.1085 PMC160915

[b18] Chalker-Scott L. Environmental significance of anthocyanins in plant stress responses. Photochem. Photobiol. 70, 1–9 (1999).

[b19] Ubi B. E. *et al.* Expression analysis of anthocyanin biosynthetic genes in apple skin: effect of UV-B and temperature. Plant Sci. 170, 571–578 (2006).

[b20] Yamane T., Jeong S., Goto-Yamamoto N., Koshita Y. & Kobayashi S. Effects of temperature on anthocyanin biosynthesis in grape berry skins. Am. J. Enol. Vitic. 57, 54–59 (2006).

[b21] Ban Y. *et al.* Isolation and functional analysis of a MYB transcription factor gene that is a key regulator for the development of red coloration in apple skin. Plant. Cell. Physiol. 48, 958–970 (2007). 17526919 10.1093/pcp/pcm066

[b22] Steyn W. J., Wand S. J. Jacobs G., Rosecrance R. C. & Roberts S. C. Evidence for a photoprotective function of low-temperatureinduced anthocyanin accumulation in apple and pear peel. Physiol. Plantarum. 136, 461–472 (2009). 10.1111/j.1399-3054.2009.01246.x 19493306

[b23] Crifo T., Petrone G., Lo Cicero L. & Lo Piero A. Short cold storage enhances the anthocyanin contents and level of transcripts related to their biosynthesis in blood oranges. J.Agric. Food .Chem. 60, 476–481 (2012). 22148517 10.1021/jf203891e

[b24] Heilmann M. & Jenkins G. I. Rapid reversion from monomer to dimer regenerates the ultraviolet-B photoreceptor UV RESISTANCELOCUS8 in intact Arabidopsis plants. Plant Physiol. 161, 547–555 (2013). 23129206 10.1104/pp.112.206805 PMC3532284

[b25] Wargent J. J., Gegas V. C., Jenkins G. I., Doonan J. H. & Paul N. D. UVR8 in *Arabidopsis thaliana* regulates multiple aspects of cellular differentiation during leaf development in response to ultraviolet B radiation. New Phytol. 183, 315–326 (2009). 19402876 10.1111/j.1469-8137.2009.02855.x

[b26] Wargent J. J., Moore J. P. , Ennos A. R. & Paul N. D. Ultraviolet radiation as a limiting factor in leaf expansion and development. Photochem. Photobiol. 85, 279–286 (2009). 18764892 10.1111/j.1751-1097.2008.00433.x

[b27] Fasano R. *et al.* Role of Arabidopsis UV RESISTANCE LOCUS 8 in plant growth reduction under osmotic stress and low levels of UV-B. Mol Plant. 7, 773–791 (2014). 24413416 10.1093/mp/ssu002

[b28] Morales L. O. *et al.* Multiple roles for UV RESISTANCE LOCUS8 in regulating gene expression and metabolite accumulation in Arabidopsis under solar UV radiation. Plant physiol. 161, 744–759 (2013). 23250626 10.1104/pp.112.211375 PMC3561016

[b29] Rizzini L. *et al.* Perception of UV-B by the Arabidopsis UVR8 protein. Science. 332, 103–106 (2011). 10.1126/science.1200660 21454788

[b30] Heijde M. & Ulm R. Reversion of the Arabidopsis UV-B photoreceptor UVR8 to the homodimeric ground state. Proc. Natl. Acad. Sci. USA. 110, 1113–1118 (2013). 23277547 10.1073/pnas.1214237110 PMC3549095

[b31] Gruber H. *et al.* Negative feedback regulation of UV-B-induced photomorphogenesis and stress acclimation in Arabidopsis. Proc. Natl. Acad. Sci. USA. 107, 20132–20137 (2010). 21041653 10.1073/pnas.0914532107 PMC2993346

[b32] Heijde M. *et al.* Constitutively active UVR8 photoreceptor variant in Arabidopsis. Proc.Natl.Acad.Sci.USA. 110, 20326–20331 (2013). 24277841 10.1073/pnas.1314336110 PMC3864333

[b33] Huang X., Yang P., Ouyang X., Chen L. & Deng X. W. Photoactivated UVR8-COP1 module determines photomorphogenic UV-B signaling output in Arabidopsis. PLOS Genetics. 10, e1004218 (2014). 24651064 10.1371/journal.pgen.1004218 PMC3961177

[b34] Kliebenstein J. D., Lim E. J., Landry G. L. & Last L. R. Arabidopsis *UVR8* regulates ultraviolet-B signal transduction and tolerance and contains sequence similarity to human Regulator of Chromatin Condensation. Plant Physiol. 130, 234–243 (2002). 12226503 10.1104/pp.005041 PMC166556

[b35] Casati P. & Walbot V. Gene expression profiling in response to ultraviolet radiation in maize genotypes with varying flavonoid content. Plant Physiol. 132, 1739–1754 (2003). 12913132 10.1104/pp.103.022871 PMC181262

[b36] Asada K. The water-water cycle in chloroplasts: scavenging of active oxygens and dissipation of excess photons. Annu. Rev. Plant. Physiol. Plant. Mol. Biol. 50, 601–639 (1999). 15012221 10.1146/annurev.arplant.50.1.601

[b37] Neill S. J., Desikan R., Clarke A., Hurst R. D. & Hancock J. T. Hydrogen peroxide and nitric oxide as signaling molecules in plants. J. Exp. Bot. 53, 1237–1247 (2002). 11997372

[b38] Zhang J. *et al.* Reactive oxygen species produced via plasma membrane NADPH oxidase regulate anthocyanin synthesis in apple peel. Planta. 240, 1023–1035 (2014). 25000919 10.1007/s00425-014-2120-4

[b39] Nikkhah E., Khaiamy M., Heidary R. & Azar A. S. The effect of ascorbic acid and H_2_O_2_ treatment on the stability of anthocyanin pigments in berries. Turk. J. Biol. 34, 47–53 (2010).

[b40] Tossi V., Lamattina L., Jenkins G. I. & Cassia R. O. Ultraviolet-B-induced Stomatal Closure in Arabidopsis is regulated by the UV RESISTANCE LOCUS8 photoreceptor in a nitric oxide-dependent mechanism. Plant Physiol. 164, 2220–2230 (2014). 24586043 10.1104/pp.113.231753 PMC3982774

[b41] He J. *et al.* Role and interrelationship of Gα protein, hydrogen peroxide, and nitric oxide in ultraviolet B-induced stomatal closure in Arabidopsis leaves. Plant Physiol. 161, 1570–1583 (2013). 23341360 10.1104/pp.112.211623 PMC3585617

[b42] Barillari J. *et al.* Isolation of 4-methylthio-3-butenyl glucosinolate from *Raphanus sativus* sprouts (kaiware daikon) and its redox properties. J. Agric. Food. Chem. 53, 9890–9896 (2005). 16366671 10.1021/jf051465h

[b43] Ciska E., Honke J. & Kozlowska H. Effect of light conditions on the contents of glucosinolates in germinating seeds of white mustard, red radish, white radish, and rapeseed. J. Agric. Food. Chem. 56, 9087–9093 (2008). 18771273 10.1021/jf801206g

[b44] Martinez-Villaluenga C., Frias J., Gulewicz P., Gulewicz K. & Vidal-Valverde C. Food safety evaluation of broccoli and radish sprouts. Food Chem and Toxicol. 46, 1635–1644 (2008). 18314243 10.1016/j.fct.2008.01.004

[b45] Barillari J. *et al.* Kaiware Daikon (*Raphanus sativus* L.) extract: A naturally multipotent chemopreventive agent. J. Agric. Food. Chem. 56, 7823–7830 (2008). 18665601 10.1021/jf8011213

[b46] Ippoushi K., Takeuchi A., Ito H., Horie H. & Azuma K. Antioxidative effects of daikon sprout (*Raphanus sativus* L.) and ginger (*Zingiber officinale* Roscoe) in rats. Food Chem. 102, 237–242 (2007).

[b47] Papi A. *et al.* Cytotoxic and antioxidant activity of 4-methylthio-3-butenyl isothiocyanate from *Raphanus sativus* L. (Kaiware Daikon) sprouts. J. Agric. Food. Chem. 56, 875–883 (2008). 18189352 10.1021/jf073123c

[b48] Wang P., Du Y. Y., Li Yuan, Ren D. T. & Song C. P. Hydrogen peroxide–mediated activation of MAP kinase 6 modulates nitric oxide biosynthesis and signal transduction in Arabidopsis. Plant Cell. 22, 2981–2998 (2010). 20870959 10.1105/tpc.109.072959 PMC2965546

[b49] Tossi V., Lamattina L., Jenkins G. I. & Cassia R. O. Ultraviolet-B-induced stomatal closure in Arabidopsis is regulated by the UV RESISTANCE LOCUS8 photoreceptor in a nitric oxide-dependent mechanism. Plant Physiol. 164, 2220–2230 (2014). 24586043 10.1104/pp.113.231753 PMC3982774

[b50] Brown B. A. *et al.* A UV-B-specific signaling component orchestrates plant UV protection. Proc. Natl. Acad. Sci. USA. 102, 18225–18230 (2005). 16330762 10.1073/pnas.0507187102 PMC1312397

[b51] Favory J. J. *et al.* Interaction of COP1 and UVR8 regulates UV-B-induced photomorphogenesis and stress acclimation in Arabidopsis. EMBO J. 28, 591–601(2009). 19165148 10.1038/emboj.2009.4 PMC2657586

[b52] Jenkins I. G. The UV-B Photoreceptor UVR8: From Structure to Physiology. Plant Cell. 26, 21–37 (2014). 24481075 10.1105/tpc.113.119446 PMC3963570

[b53] Sgherri C., Scattino C., Pinzino C., Tonutti P. & Ranieri A. M. Ultraviolet-B radiation applied to detached peach fruit: A study of free radical generation by EPR spin trapping. Plant Physiology and Biochemistry. 96, 124–131 (2015). 26263515 10.1016/j.plaphy.2015.07.031

[b54] Josuttis M. *et al.* Solar UVB response of bioactives in strawberry (*Fragaria ananassa* Duch. L.): A comparison of protected and open-field cultivation. J. Agric. Food Chem. 58, 12692–12702 (2010). 21086998 10.1021/jf102937e

[b55] Tsormpatsidis E. *et al.* UV irradiance as a major influence on growth, development and secondary products of commercial importance in Lollo Rosso lettuce ‘Revolution’ grown under polyethylene films. Environ Exp Bot. 63, 232–239 (2008).

[b56] Mao K., Wang L., Li Y. & Wu R. Molecular cloning and functional analysis of UV RESISTANCE LOCUS 8 (*PeUVR8*) from *Populus euphratica*. PLOS ONE. e0132390 (2015). 26171608 10.1371/journal.pone.0132390 PMC4501546

[b57] Page M., Sultana N., Paskiewicz K., Florance H. & Smirnoff N. The influence of ascorbate on anthocyanin accumulation during high light acclimation in *Arabidopsis thaliana*: further evidence for redox control of anthocyanin synthesis. Plant Cell. Environ. 35, 388–404 (2012). 21631536 10.1111/j.1365-3040.2011.02369.x

[b58] Bai S. L. *et al.* An apple B-box protein, MdCOL11, is involved in UV-B- and temperature-induced anthocyanin biosynthesis. Planta. 240, 1051–1062 (2014). 25074586 10.1007/s00425-014-2129-8

[b59] Rubin G., Tohge T., Matsuda F., Saito K. & Scheible W. R. Members of the *LBD* family of transcription factors repress anthocyanin synthesis and affect additional nitrogen responses in Arabidopsis. Plant Cell. 21, 3567–3584 (2009). 19933203 10.1105/tpc.109.067041 PMC2798321

[b60] Xie Y. J. *et al.* Reactive oxygen species-dependent nitric oxide production contributes to hydrogen-promoted stomatal closure in Arabidopsis. Plant Physiol. 165, 759–773 (2014). 24733882 10.1104/pp.114.237925 PMC4044830

[b61] Dulak J. & Jozkowicz A. Carbon monoxide–a new gaseous modulator of gene expression. Acta Biochim Pol. 50, 31–47 (2003). 12673345

[b62] Crawford N. M. *et al.* Response to Zemojtel *et al.*: plant nitric oxide synthase: back to square one. Trends in Plant Science. 11, 526–527 (2006). 10.1016/j.tplants.2006.09.008 17030145

[b63] Zemojtel T. *et al.* Plant nitric oxide synthase: a never-ending story? Trends in Plant Science. 11, 524–525 (2006). 17030145 10.1016/j.tplants.2006.09.008

[b64] Sudhamsu J., Lee G. I., Klessig D. F. & Crane B. R. The structure of YqeH. An AtNOS1/AtNOA1 ortholog that couples GTP hydrolysis to molecular recognition. J Bio Chem. 283, 32968–32976 (2008). 18801747 10.1074/jbc.M804837200 PMC2583316

[b65] Zhao M. G., Tian Q. Y. & Zhang W. H. Nitric oxide synthasedependent nitric oxide production is associated with salt tolerance in Arabidopsis. Plant Physiol. 144, 206–217 (2007). 17351048 10.1104/pp.107.096842 PMC1913813

[b66] Mandal M. K. *et al.* Oleic acid dependent modulation of NITRIC OXIDE ASSOCIATED1 protein levels regulates nitric oxide-mediated defense signaling in Arabidopsis. Plant Cell. 24, 1654–1674 (2012). 22492810 10.1105/tpc.112.096768 PMC3398570

[b67] Rockel P., Strube F., Rockel A., Wildt J. & Kaiser W. M. Regulation of nitric oxide (NO) production by plant nitrate reductase *in vivo* and *in vitro*. J Exp Bot. 53, 103–110 (2002). 11741046

[b68] Gupta K. J., Fernie A. R., Kaiser W. M. & van Dongen J. T. On the origins of nitric oxide. Trends in Plant Science. 16, 160–168 (2011). 21185769 10.1016/j.tplants.2010.11.007

[b69] Jenkins G. I. The UV-B photoreceptor UVR8: from structure to physiology. Plant Cell. 26, 21–37 (2014). 24481075 10.1105/tpc.113.119446 PMC3963570

[b70] Christie J. M. *et al.* Plant UVR8 photoreceptor senses UV-B by tryptophan-mediated disruption of cross-dimer salt bridges. Science. 335, 1492–1496 (2012). 22323738 10.1126/science.1218091 PMC3505452

[b71] Wu D. *et al.* Structural basis of ultraviolet-B perception by UVR8. Nature. 484, 214–219 (2012). 22388820 10.1038/nature10931

[b72] Rizzini L. *et al.* Perception of UV-B by the Arabidopsis UVR8 protein. Science. 332, 103–106 (2011). 10.1126/science.1200660 21454788

[b73] Brown B. A. & Jenkins G. I. UV-B signaling pathways with different fluence-rate response profiles are distinguished in mature Arabidopsis leaf tissue by requirement for UVR8, HY5, and HYH. Plant Physiol. 146, 576–588 (2008). 18055587 10.1104/pp.107.108456 PMC2245850

[b74] Zhou B., Guo Z. F., Xing J. P. & Huang B. R. Nitric oxide is involved in abscisic acid-induced antioxidant activities in Stylosanthes guianensis. J. Exp. Bot. 56, 3223–3228 (2005). 16263901 10.1093/jxb/eri319

[b75] Sun C. *et al.* Nitrate reductase-mediated early nitric oxide burst alleviates oxidative damage induced by aluminum through enhancement of antioxidant defenses in roots of wheat (*Triticumaestivum*). New Phytol. 201, 1240–1250 (2014). 24237306 10.1111/nph.12597

[b76] González A. *et al.* Cross talk among calcium, hydrogen peroxide, and nitric oxide and activation of gene expression involving calmodulins and calcium-dependent protein kinases in Ulva compressa exposed to copper excess. Plant Physiol. 158, 1451–1462 (2012). 22234999 10.1104/pp.111.191759 PMC3291273

[b77] Hung K. T., Cheng D. G., Hsu Y. T. & Kao C. H. Abscisic acid-induced hydrogen peroxide is required for anthocyanin accumulation in leaves of rice seedlings. J. Plant Physiol. 165, 1280–1287 (2008). 18160127 10.1016/j.jplph.2007.10.008

[b78] Hu X., Fang J., Cai W. & Tang Z. NO-mediated hypersensitive responses of rice suspension cultures induced by incompatible elicitor. Chinese. Sci. Bull. 48, 358–363 (2003).

