# Supplementary material for: Hydrogen peroxide, nitric oxide and UV RESISTANCE LOCUS8 interact to mediate UV-B-induced anthocyanin biosynthesis in radish sprouts
Source: Sci Rep. 2016 Jul 12;6:29164. doi: 10.1038/srep29164 (PMC4941517; doi:10.1038/srep29164)
Supplement: Supplementary Information [file srep29164-s1.pdf]

Hydrogen peroxide, nitric oxide and UV RESISTANCE LOCUS8 interact to mediate UV-B-induced anthocyanin biosynthesis in radish sprouts

Qi Wu, Nana Su, Xiaoyan Zhang, Yuanyuan Liu, Jin Cui, Yongchao Liang

Supplementary Table S1. The nucleotide sequence of primers used in the RT-PCR

cDNA	Primer forward	Primer reverse
Actin	5' GCTCAGTCCAAGAGAGGTATTC 3'	5'GCTCGTTGTAGAAAGTGTGATG 3'
PAL	5'GAATTTAACCGCTTCCAACA3'	5'AAGCTCAGAGCAGTAAGAAG 3'
CHS	5'CTTGACCGAAGAGTTCTTGA 3'	5'GTCTTGTCTAGCATCGAGAG 3'
CHI	5'AACGTTCCCTCCTGGTGCTT 3'	5'TTTTCCCGTTTCAGGAATGC 3'
F3H	5'CAAAATGCCCTCAGCCTGAT 3'	5'GCTTGTAAACCACCGACTTGGT 3'
DFR	5'GGATCTGCAGGTTTAACTGA 3'	5'TGCGACTATCTGTTTTCTCG 3'
LDOX	5'GTTTGCAGCTTTTCTACGAGG3'	5'TGAGCAAAAGTCCGTGGAGG3'
ANS	5'ACGTAAGCGCTTTGACCTTCA 3'	5'ATCGAATCGGGAACGCATT 3'
UFGT	5'TGTCAGATCGTTTTGGTTCC3'	5'GATTCTTCCTCACTTTCTCAC3'
UVR8	5'ATCCCCAGTGAGTAGAGCGA3'	5'TGATCGATGGCTGAAGAGGAG3'
NR	5' CGTGAGAAAGTCCCGGTGAA3'	5' TAGTGGCGCAGAGGAAGATG3'
NOA1	5'GAACCAATCCGCAGAGTACG3'	5'CCATTCAGCACCAGAAGTGC3'
COP1	5' TCAGCTGACCATCACATCC3'	5' ACCGCTTTCTTGTGTCCACT3'
PAP1	5'TGAGTAAGAAACATGAACCAGGC 3'	5'GCCGTTGTTAACCGTGAAGGA 3'
PAP2	5'ATGGAGAAGGCAAATGGCATCAAG3'	5'CCAGCAATCAAGGACCACCTATTTC3'

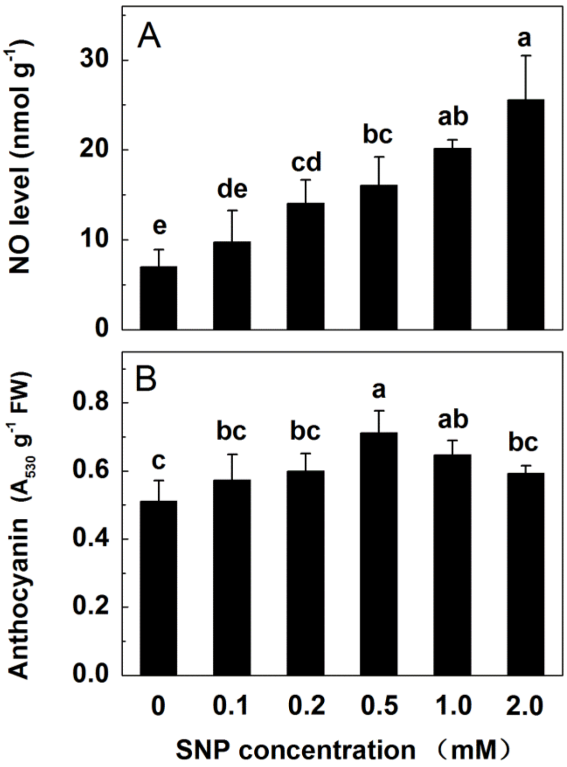

Supplementary Figure S1. Effects of exogenous addition of increasing SNP concentrations on the endogenous NO level (A) and anthocyanin accumulation (B). After 24 h dark incubation, the radish sprouts were subjected to SNP with different concentrations for 12 h, and were then exposed to white light for another 24 h. The data are means ± SD of three independent experiments. Significance between experimental values was assessed by Duncan's test ($P< 0.05$).

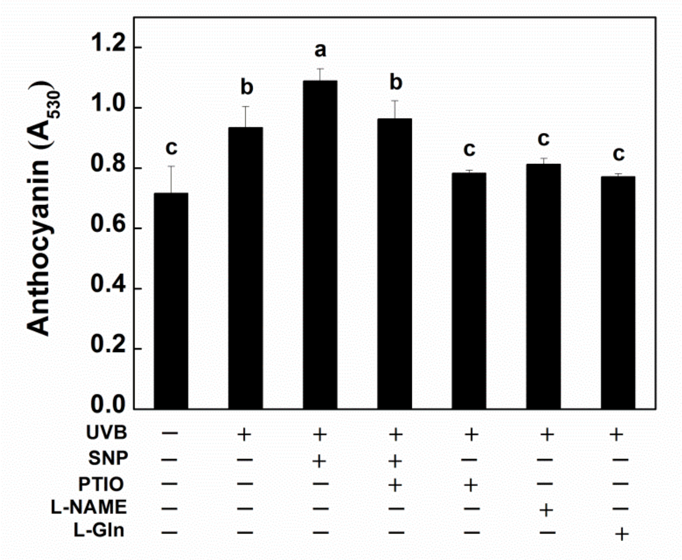

Supplementary Figure S2. Effects of different combinations of UV-B, SNP, PTIO, L-NAME and L-Gln on the anthocyanin accumulation in the hypocotyls of radish sprouts. After 36 h dark incubation, the radish sprouts were subjected to different treatments for another 36 h, and the hypocotyls were then collected for measurement. The data are means ± SD of three independent experiments. Significance between experimental values was assessed by Duncan's test (P< 0.05).

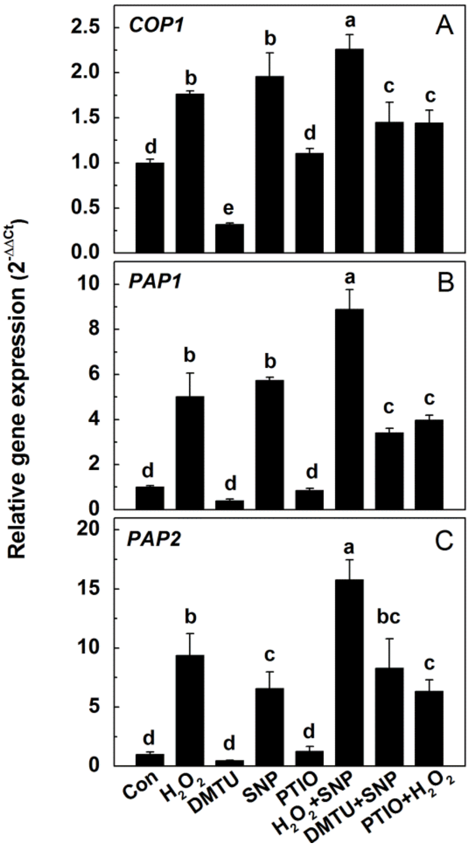

Supplementary Figure S3. The transcript levels of *COP1* (A), *PAP1* (B) and *PAP2* (C) under different treatments. After 36 h dark incubation, the radish sprouts were then subjected to H_2O_2, DMTU, SNP, PTIO or their combination under white light for 24 h. The data are means ± SD of three independent experiments. Significance between experimental values was assessed by Duncan's test ($P < 0.05$).

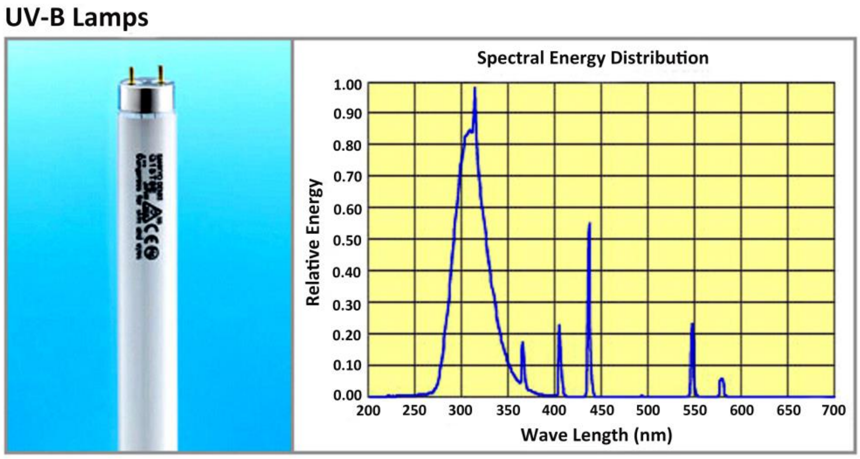

Supplementary Figure S4. The spectral energy distribution of UV-B lamps.
